# Mitochondrial Biogenesis in Neurons: How and Where

**DOI:** 10.3390/ijms222313059

**Published:** 2021-12-02

**Authors:** Carlos Cardanho-Ramos, Vanessa Alexandra Morais

**Affiliations:** Instituto de Medicina Molecular—João Lobo Antunes, Faculdade de Medicina, Universidade de Lisboa, 1649-028 Lisbon, Portugal; carlos.ramos@medicina.ulisboa.pt

**Keywords:** mitochondrial biogenesis, neurons, cell body, periphery, PGC-1α, NRF-1/2, TFAM, neurodegenerative diseases

## Abstract

Neurons rely mostly on mitochondria for the production of ATP and Ca^2+^ homeostasis. As sub-compartmentalized cells, they have different pools of mitochondria in each compartment that are maintained by a constant mitochondrial turnover. It is assumed that most mitochondria are generated in the cell body and then travel to the synapse to exert their functions. Once damaged, mitochondria have to travel back to the cell body for degradation. However, in long cells, like motor neurons, this constant travel back and forth is not an energetically favourable process, thus mitochondrial biogenesis must also occur at the periphery. Ca^2+^ and ATP levels are the main triggers for mitochondrial biogenesis in the cell body, in a mechanism dependent on the Peroxisome-proliferator-activated γ co-activator-1α-nuclear respiration factors 1 and 2-mitochondrial transcription factor A (PGC-1α-NRF-1/2-TFAM) pathway. However, even though of extreme importance, very little is known about the mechanisms promoting mitochondrial biogenesis away from the cell body. In this review, we bring forward the evoked mechanisms that are at play for mitochondrial biogenesis in the cell body and periphery. Moreover, we postulate that mitochondrial biogenesis may vary locally within the same neuron, and we build upon the hypotheses that, in the periphery, local protein synthesis is responsible for giving all the machinery required for mitochondria to replicate themselves.

## 1. Introduction

Mitochondria are two-membrane organelles that contain their own DNA and can replicate independently of the host cell. As the powerhouse of the cell, mitochondria are responsible for ATP production through oxidative phosphorylation (OxPhos). Nonetheless, mitochondria also play a central role in Ca^2+^ homeostasis, redox regulation, fatty acid synthesis and oxidation, and apoptosis [1]. Neurons are high energy demanding cells and require a tight regulation of Ca^2+^ for the maintenance of their action potentials. Thus, it comes as no surprise that mitochondrial malfunction in neurons leads to degenerative diseases [2]. Morphologically, neurons are composed by a cell body, dendrites, axons and synapses. These different compartments have different functions and require specific pools of mitochondria [3,4,5]. To achieve this, mitochondria are constantly being transported back and forth; they fuse, divide and replicate, and when damaged they are degraded by a specific autophagy process named mitophagy [6]. All these processes are interconnected and have to be finely tuned in order to maintain a healthy, robust pool of neurons, hence preventing neurodegenerative diseases.

This constant movement of mitochondria in neurons has been associated with several proteins. Anterograde transport is mediated by the Kinesin-1 family (KIF5) [7,8], which binds to mitochondria through TRAK [9] and Mitochondrial Rho GTPase (Miro) [10], or through syntabulin [8]. Dynein and Dynactin play a crucial role in the retrograde transport [7,11]. Although Dynein can bind directly to mitochondria [12], Miro may also be involved [13]. Mitochondria transport is then halted in regions with high ATP demands [14,15] and Ca^2+^ homeostasis [16], such as synapses. Mitochondria can also fuse and divide. While fusion is essential to rescue defective mitochondria through the cross-exchange of intrinsic proteins, lipids and mitochondrial DNA (mtDNA), fission is required for the distribution of mitochondria within the cell. Mitofusin 1 and 2 (Mfn1 and Mfn2), and Optic Atrophy 1 (OPA-1) are required for fusion of the outer and inner mitochondrial membranes, respectively [17], whereas Dynamin-related protein 1 (Drp1), mitochondrial fission protein 1 (Fis1), and mitochondrial fission factor (MFF) are responsible for fission [18]. However, when damage is irreversible mitochondria undergo a selective clearance pathway named mitophagy [19]. Although mitophagy has been observed locally in axons [20], it is still assumed that mitochondria must travel to the cell body for complete degradation [21,22,23]. Maintaining a healthy pool of mitochondria within a cell requires all these processes, perhaps the most important one being mitochondrial biogenesis. Mitochondrial biogenesis can be defined as a set of molecular processes that ultimately result in mitochondrial replication. Since mtDNA only encodes for 13 proteins, the remaining over 1000 mitochondrial resident proteins are all nuclear-encoded [24]. This implies a constant communication between the nucleus and mitochondria. Indeed, in response to certain stimuli, such as thermogenic regulation and exercise, the expression of Peroxisome-proliferator-activated γ co-activator-1α (PGC-1α) is induced. PGC-1α is the master regulator of mitochondrial biogenesis. It activates nuclear respiration factors 1 and 2 (NRF-1 and NRF-2), leading to the expression of several mitochondrial genes, including proteins that are required for mtDNA transcription and replication, namely mitochondrial transcription factor A (TFAM) (reviewed in [25,26]). Thus, it comes as no surprise that PGC-1α-null mice present structural lesions in the brain and behavioural changes characteristic of neurological disorders [27]. In neurons, it is thought that most mitochondria are generated in the cell body and then travel to the locations where they are required; however, mitochondrial biogenesis has been observed to occur in the periphery [28,29]. Interestingly, the same signals that anchor mitochondria at synapses, such as depletion of ATP or increased levels of Ca^2+^, also induce mitochondrial biogenesis [30,31]. Whether these mechanisms are responsible for mitochondrial biogenesis in distal axons or dendrites is still unknown. Understanding the regulation of mitochondrial biogenesis in neurons may unravel a new therapeutic target to improve neuronal health and delay the development of neurodegenerative diseases.

## 2. Mechanisms for Mitochondrial Biogenesis

mtDNA is a double stranded molecule of 16.6 kb, encoding 13 subunits of the OxPhos system, 2 ribosomal RNAs and 22 transfer RNAs. mtDNA also has a noncoding region, the D-loop, which contains promoters and the origin of replication. In mitochondria, replication is tightly connected to transcription. For mtDNA replication to occur, the mitochondrial RNA polymerase (POLRMT) is required to form an RNA primer, and then replication is carried out by the DNA polymerase γ (POLγ), assisted by the DNA helicase TWINKLE and by the mitochondrial single-stranded DNA-binding protein (mtSSB), which protects single-stranded DNA against nucleases and prevents the formation of secondary structures (reviewed in [32]). TFAM plays a pivotal role in mtDNA replication, as it binds upstream of the transcription start site, folding the DNA and allowing the recruitment of POLRMT. mtDNA is also associated with TFAM for packaging in nucleoids. More compact nucleoids represent a mtDNA storage form, whereas looser forms are involved in active replication and transcription. Therefore, mitochondrial biogenesis is highly dependent on TFAM function.

Mitochondrial biogenesis is regulated by the PGC-1α-NRF-1/2-TFAM pathway. PGC-1α was initially observed to be increased in thermogenic tissues in response to the cold, which in turn led to the expression of several genes from the respiratory chain and an increase in mtDNA content [33]. Gain of function experiments in both cultured cells [34] and transgenic mice [35] supported this role. In myotubes, ectopic expression of PGC-1α increases mtDNA and the mitochondrial number [34]. Cardiac-specific overexpression in transgenic animals results in a massive increase in mitochondrial content [35]. PGC-1α also promotes mitochondrial function as it induces the expression of OxPhos subunits, which stimulates mitochondrial respiration [34,35]. Although with multi-system abnormalities, PGC-1α-null mice are viable [27,36]. Interestingly, TFAM is reduced in the skeletal muscle of PGC-1α-null mice which co-relates with a decrease in exercise tolerance [36]. Mice lacking PGC-1α also present an abnormal brain morphology, particularly in the striatum, where neurite growth is impaired [27]. This results in hyperactivity, among other behavioural changes. PGC-1α, albeit indirect, is undoubtedly crucial for mitochondrial replication. NRF-1 and NRF-2 bind to the promoter region of many mitochondrial genes, including those induced by overexpression of PGC-1α, such as TFAM. Indeed, it has been observed that PGC-1α activates NRF-1 and NRF-2, and that a double negative allele of NRF-1 blocks PGC-1α effects on mitochondrial biogenesis [37]. Thus, NRF-1 and NRF-2 act downstream of PGC-1α, regulating not only genes involved in OxPhos, but also TFAM, resulting in increased mitochondrial respiration and mtDNA replication/transcription.

**Figure 1:**
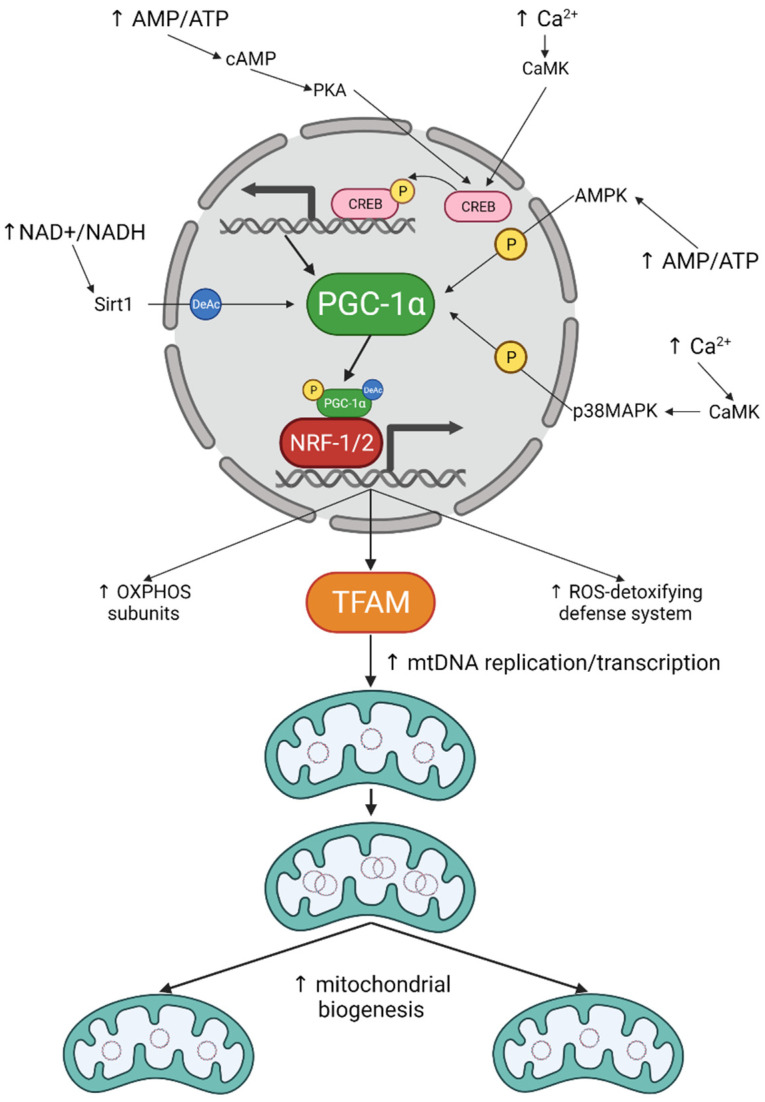
Mitochondrial biogenesis signalling cascades. Ca^2+^ and AMP/ATP are the main stimuli for mitochondrial biogenesis. Elevated Ca^2+^ and AMP levels activate different kinases that can either phosphorylate CREB to promote PGC-1α expression; or directly phosphorylate PGC-1α leading to its activation. Increased NAD^+^/NADH ratio also results in PGC-1α activation through deacetylation by Sirt1. Once activated, PGC-1α binds to NRF-1/2 to promote expression of several mitochondrial proteins, including TFAM, ultimately resulting in increased mitochondrial biogenesis.

Several signalling cascades activate mitochondrial biogenesis through the PGC-1α-NRF-1/2-TFAM pathway (Figure 1). From those, AMP/ATP ratio and Ca^2+^ levels are the most relevant. Increased levels of AMP activate adenosine monophosphate protein kinase (AMPK), which directly phosphorylates PGC-1α [30]. Phosphorylated PGC-1α is then able to regulate its own gene expression, as well as several mitochondrial genes [30]. AMPK has also been observed to induce mitochondrial biogenesis through NRF-1 [38]. This also probably results from the direct activation of PGC-1α by AMPK. Recently, it was reported that AMPK phosphorylated epigenetic factors, leading to increased expression of PGC-1α and TFAM [39]. AMP can also be converted to cyclic AMP (cAMP) by adenyl cyclase (AC). cAMP triggers many cellular pathways, namely through the cAMP-dependent kinase (PKA). In the nucleus, PKA phosphorylates the cAMP response element binding (CREB) protein [40], a transcription factor that binds to the PGC-1α promoter [41]. Activation of AC by forskolin increased cAMP levels which ultimately resulted in the elevation of PGC-1α. This was abrogated when a dominant negative form of CREB was expressed [42]. cAMP-PKA-CREB pathway modulated PGC-1α, leading to the activation of NRF-1/2 and TFAM, thus promoting mitochondrial biogenesis (Figure 1). cAMP, PKA and CREB are also present inside mitochondria, not to directly promote mitochondrial biogenesis, but to modulate OxPhos subunits and mitochondrial respiration [43,44].

Ca^2+^ is also an important regulator of mitochondrial biogenesis. Increasing Ca^2+^ levels induce the expression of PGC-1α and TFAM [45]. This was accompanied by an increase in NRF-1 and NRF-2 binding to DNA [45]. The effect of Ca^2+^ on mitochondrial biogenesis was abolished by blocking either the calcium/calmodulin-dependent protein kinase (CaMK) [31,45] or the p38 mitogen-activated protein kinase (p38 MAPK) [31]. p38 MAPK is a downstream target of CaMK and has been seen to regulate PGC-1α activation and expression [46]. Thus, Ca^2+^ stimulates CaMK, which in turn phosphorylates p38MAPK, leading to the activation and expression of PGC-1α, ultimately resulting in increased mitochondrial biogenesis (Figure 1). Additionally, CaMK has also been shown to stimulate PGC-1α through CREB [42]. This implies that CREB may play a role in the Ca^2+^-dependent mitochondrial biogenesis.

Another important signalling for mitochondrial biogenesis is the NAD^+^/NADH ratio. In response to NAD^+^, Sirtuin 1 (Sirt1) deacetylates PGC-1α leading to its activation [47] (Figure 1). Although deacetylation of PGC-1α is only described as stimulating fatty acid oxidation, one could speculate that it may also play a role in mitochondrial biogenesis. Notably, it seems that deacetylation of PGC-1α leads to an increase in mitochondrial content, in a mechanism dependent on Ca^2+^, AMPK and Sirt1 [48]. This implies that all the major mitochondrial biogenesis stimuli may be interconnected.

Other stimuli, such as reactive oxygen species (ROS), also promote PGC-1α expression. This is particularly important for the ROS-detoxifying defence system, once PGC-1α mediate the expression of superoxide dismutase (SOD1 and SOD2), catalase and glutathione peroxidase (GPx1).

## 3. Mitochondrial Biogenesis in Neurons: The How

Neurons are complex cells, with multiple compartments, that can last a lifetime. Mitochondria, however, need to be constantly renovated whether they are in the cell body, dendrites, axon or synapse. This leads us to speculate if the mechanisms that regulate mitochondrial biogenesis are the same in these different subcellular compartments.

**Figure 2:**
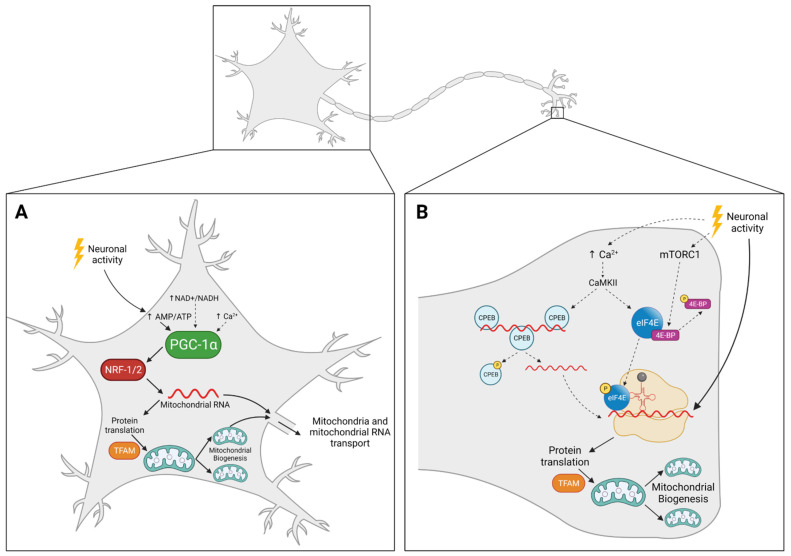
Possible mechanisms for mitochondrial biogenesis in neurons: cell body vs. periphery. (**A**) Neuronal activity activates PGC-1α through increased AMP/ATP ratio and AMPK. Activation of PGC-1α leads to increased mitochondrial RNA that can either be used for protein translation and mitochondrial renewal or be directly transported to the periphery. Ca^2+^ and NAD^+^/NADH ratio may also be involved. (**B**) Neuronal activity promotes mitochondrial protein translation and mitochondrial biogenesis at the periphery. Although the mechanism is not completely understood, we hypothesized that it may be dependent on Ca^2+^ and mTORC1. Both Ca^2+^ and mTORC1 have been seen to increase protein translation in the periphery, whether or not it promotes mitochondrial biogenesis is still unclear. Full arrows represent mechanisms already described in neurons, whereas dashed arrows represent hypothetical mechanisms proposed by the authors.

As in other cells, PGC-1α is the master regulator of mitochondrial biogenesis in neurons. PGC-1α regulates mitochondrial density in axons, in a mechanism dependent on Sirt1 [49] (Figure 2A). The depolarization of neurons activates AMPK, leading to increased expression levels of PGC-1α, NRF-1 and TFAM, resulting in augmented mitochondrial content and ATP levels in cultured primary neurons [50] (Figure 2A). This observation suggests that mitochondrial biogenesis is tightly connected to synaptic activity (Figure 2). Indeed, knockdown of PGC-1α results in a marked decrease of dendritic mitochondria, leading to the reduction of the number of synapses [51]. This effect was observed in both embryonic neurons and adult brains, suggesting that PGC-1α plays a critical role not only in the formation, but also in the maintenance of synapses. These results are in line with the observation that mitochondria are required for the formation of filopodia and maturation into axon branches [52]. Additionally, the brain derived neurotrophic factor (BDNF)-induced synaptic plasticity was suppressed by knocking down PGC-1α. BDNF induces CREB phosphorylation, leading to elevated levels of PGC-1α, NRF-1 and TFAM, and, subsequently, in increased mitochondrial biogenesis and function [51]. Together, these results confirm that PGC-1α and mitochondrial biogenesis have an important role in neuronal activity.

The importance of mitochondrial biogenesis in neurons is also reflected by the observation that PGC-1α, NRF-1 and TFAM are downregulated in several neurodegenerative diseases, such as Huntington’s (HD), Parkinson’s (PD) and Alzheimer’s diseases (AD) (reviewed in [53]).

HD is characterized by a mutation in the *huntingtin* gene, leading to the accumulation of mutant huntingtin and the degeneration of neurons, particularly in the striatum. Mutant huntingtin has been observed to bind to the PGC-1α promoter, repressing its transcription [54]. Accordingly, PGC-1α expression is reduced specifically in the striatum of HD patients, and restoring PGC-1α in a mouse model of HD rescued neuronal loss [54]. As mentioned above, PGC-1α KO mice also presented lesions in the striatum and the abnormal behaviour that resembles HD [55]. These results suggest that downregulation of PGC-1α and hampered mitochondrial biogenesis are at the base of HD pathology.

PD patients show lower levels of mtDNA [56], as well as PGC-1α and its target genes [57]. Decreased level of PGC-1α has been proposed to be due to the accumulation of the parkin interacting substrate (PARIS) [58,59]. PARIS is able to bind to the PGC-1α promoter, repressing its activity and downregulating PGC-1α and NRF-1, leading to dopaminergic neuronal loss [59]. Although PARIS degradation is mediated by Pink1 and Parkin [60], two proteins associated with familial forms of PD, PARIS accumulation has also been observed in sporadic PD [59]. Specifically disrupting TFAM in dopaminergic neurons also led to a decrease in mtDNA levels and respiratory function, consequently resulting in neuronal death. This was accompanied by decreased locomotion and an increased tremor, a phenotype characteristic of PD [61].

AD brains present a decreased mitochondrial number [62] and lower levels of PGC-1α, NRF-1/2 and TFAM [63]. Furthermore, an AD cell line presented defects in mitochondrial biogenesis and respiration, but these phenotypes were completely rescued by the overexpression of PGC-1α [63].

Increasing evidence indicates that neurodegenerative diseases may result from hampered mitochondrial biogenesis, particularly at the level of PGC-1α. This connection may open a new avenue for the development of novel therapeutic strategies.

## 4. Mitochondrial Biogenesis in Neurons: The Where

The mitochondrial proteome is composed of more than 1000 proteins, from which only 13 are mitochondrial-encoded [24]. The fact that mitochondria require so many nuclear-encoded proteins led to the assumption that mitochondrial biogenesis in neurons is restricted to the cell body. This view has been challenged by the observation that mtDNA replication also occurs distally in axons [28,29].

The question of where mitochondrial biogenesis occurs has been extensively discussed [64,65], and two main models have emerged: (1) mitochondria replicate in the cell body and travel back and forth to supply distal regions; (2) mitochondria replicate locally, away from the cell body.

### 4.1. Mitochondria Replicate in the Cell Body and Travel Back and Forth to Supply Distal Regions

In this model, mitochondria replicate at the cell body and then travel to distal regions, such as synapses, where they will replace damaged mitochondria that in turn travel back to the cell body for degradation. This model is supported by the fact that mtDNA only encodes for proteins that take part in the OxPhos system, which means all the machinery required for mtDNA replication is nuclear-encoded. Additionally, as seen above, all the mitochondrial biogenesis pathways known so far require PGC-1α and NRF-1/2, and subsequently the activation of the nuclear transcription (Figure 2A). If mitochondrial biogenesis was to occur distally, it would imply a mechanism that does not involve the master regulator PGC-1α. Giving the importance of mitochondrial biogenesis and PGC-1α in the formation and maintenance of synapses, as well as in the pathophysiology of neurodegenerative diseases, such a mechanism seems unlikely.

In neurons, around 30% of mitochondria are constantly travelling back and forth; however, the motile pool of mitochondria is not always the same. Stationary mitochondria have been observed to acquire movement even after long periods of pause, whereas moving mitochondria anchor at regions with high levels of Ca^2+^ or high ATP demands. Thus, mitochondria know when and where to move, supporting the model where newly formed mitochondria at the cell body have the ability to sense where they are needed and when they should be transported to the periphery. One problem of this model is the transport velocity of mitochondria in neurons, which is estimated to be around 0.5 µm/s [66,67]. These estimations also apply to motor neurons [68], meaning that in these neurons a newly formed mitochondrion would take several days to go from the cell body to the tip of the axon. Additionally, as primary neurons mature, the percentage of moving mitochondria decreases to levels <10% [69], similar to what is observed in vivo [70,71].

This model may be sufficient to explain mitochondrial biogenesis in short neurons or in small animals, but when we consider motor neurons, that in humans can be 1m long, it is unreasonable to accept that mitochondrial renewal only occurring within the cell body is the only model in place.

### 4.2. Mitochondria Replicate Locally, Away from the Cell Body

If the mitochondrial transport rate is too slow for neurons to rely only on mitochondrial biogenesis in the cell body, mitochondrial biogenesis must also take place distally. Accordingly, mtDNA replication has been seen to occur away and independently from the cell body [28,29]. Renewal of mitochondria requires nuclear transcription activation, mediated by PGC-1α and NRF1/2. Since PGC-1α and NRF1/2 only act in the nucleus, distal mitochondria require an alternative mechanism to have access to newly synthesized proteins. This issue can be overcome by the presence of mRNA for mitochondrial proteins in axons [72,73] and by the observation of axonal translation of both mitochondrial- [74] and nuclear-encoded [75,76,77] proteins. Instead of constantly transporting mitochondria from the cell body to the periphery, mRNAs, that can be translated several times, are present distally, providing all the machinery necessary for mitochondria to replicate themselves away from the cell body (Figure 2B). This model is supported by several reports where inhibiting local protein synthesis led to mitochondrial dysfunction in axons [75,78].

Neuronal activity stimulates local protein translation, essential for synaptic plasticity and memory [79,80], in a process fuelled by mitochondria [81]. Mitochondrial protein synthesis is also enhanced by synaptic activity [74,76]. Together, these observations suggest that neuronal activity may be the trigger to induce distal mitochondrial biogenesis (Figure 2B). Mechanistically, this could be achieved by Ca^2+^ activation of CaMKII. This is supported by the fact that CaMKII phosphorylates cytoplasmic polyadenylation element binding protein (CPEB) in hippocampal neurons [82]. CPEB is an RNA-binding protein that, when phosphorylated, stimulates mRNA polyadenylation to initiate translation (Figure 2B). CaMKII has also been observed to activate eukaryotic translation initiation factor 4E (eIF4E), leading to increased protein synthesis at the synapse [83] (Figure 2B). Another possible mechanism involves mammalian target of the rapamycin complex 1 (mTORC1). mTORC1 also activates eIF4E and has been reported to induce translation at the synapse [84] (Figure 2B). Interestingly, AMPK counteracts the mTORC1-induced protein synthesis in neurons [85], suggesting that AMPK may inhibit mitochondrial biogenesis away from the cell body.

## 5. Conclusions and Future Perspectives

Mitochondrial dysfunction is at the base of neurodegeneration. For each disease a specific mechanism has been hypothesized to explain how mitochondrial malfunction leads to neuronal loss. Defects in mitochondrial biogenesis, however, are characteristic of most neurodegenerative diseases, suggesting that the inability to replicate correctly is the common denominator leading to defective mitochondria. Future studies should focus more on unravelling mitochondrial biogenesis mechanisms in neurons, particularly by confirming if the results obtained in cell lines and muscle also apply. It is important to keep in mind that there are multiple neuronal sub-compartments, with specific mitochondrial pools. Furthermore, neurons can be morphologically and functionally very different from each other; there are sensory neurons, interneurons, motor neurons, and some are short while others have really long axons. Therefore, mitochondrial biogenesis mechanisms may differ from neuron to neuron, and multiple mechanisms may take place within the same cell depending on location. In small neurons, the model where mitochondria replicate in the cell body and then travel back and forth may be sufficient to maintain a healthy pool of mitochondria across the entire neuron. Nevertheless, neurons with long axons, most probably also present mitochondrial biogenesis away from the cell body. It will be important to discriminate these different mechanisms and access their individual impact in neurodegeneration. One could speculate that in the early stages, where only synaptic defects are observed, distal mitochondrial biogenesis has a bigger impact, whereas defective mitochondrial replication in the cell body leads to a more permanent damage, such as neuronal loss. Understanding the different mechanisms of mitochondrial biogenesis in neurons and how they are regulated will give us new insights on neuronal physiology and maintenance of mitochondrial fitness at the synapse, and ultimately may lead to a better comprehension with regards to the development of new therapies for several neurological disorders.
